# Gardner's syndrome in a 40-year-old woman: successful treatment of locally aggressive desmoid tumors with cytotoxic chemotherapy

**DOI:** 10.1186/1477-7819-4-96

**Published:** 2006-12-17

**Authors:** Prabhat K Bhama, Rashmi Chugh, Laurence H Baker, Gerard M Doherty

**Affiliations:** 1 Department of Surgery, Division of Endocrine Surgery, University of Michigan, Ann Arbor, Michigan, USA; 2 Department of Internal Medicine, Division of Hematology-Oncology, University of Michigan, Ann Arbor, Michigan, USA

## Abstract

**Background:**

Desmoid tumors that present as a part of Gardener's syndrome can present very difficult management problems.

**Case presetation:**

We report a case of intra-abdominal desmoid tumor causing distal small bowel obstruction that complicated the management of a more proximal enterocutaneous fistula from the jejunum. After failure of more conventional management options including imatinib, the patient's disease responded to doxorubicin and ifosfamide. The response resolved the bowel obstruction and allowed small intestinal resection to resolve the enterocutaneous fistula.

**Conclusion:**

Systemic cytotoxic therapy with doxorubicin and ifosfamide can be useful for patients with complications from intra-abdominal desmoid tumor.

## Background

Gardner's syndrome includes familial adenomatous polyposis with extracolonic manifestations, such as osteomas, supernumerary teeth, fibrous dysplasia of the skull, fibromas, and epidermoid cysts. Desmoid tumors, also known as "aggressive fibromatosis", can have a significant impact on the morbidity and mortality of patients with Gardner's syndrome. Locally aggressive desmoid tumors can impinge on surrounding structures, causing symptoms and organ impairment. When treatment is indicated, these tumors should be surgically resected with wide margins. However, resection is often followed by recurrence, and resection of some lesions would cause unacceptable deficits. Although desmoid tumors have no metastatic potential, systemic therapy with a variety of agents can be helpful. Cytotoxic chemotherapy is effective in some cases refractory to more conservative treatment options. We report a case of a 40 year-old woman with Gardner's Syndrome and desmoid tumors causing an enterocutaneous fistula with distal small bowel obstruction, successfully managed with a strategy that included cytotoxic chemotherapy.

## Case presentation

A 40-year-old-woman with a history of Gardner's Syndrome status post prophylactic colectomy presented with an enterocutaneous fistula and for reevaluation of desmoid tumors. She had three previous abdominal operations including a prophylactic colectomy in 1992. In 1995, she had a resection of small intestine and marginal resection of associated desmoid tumors causing small bowel obstruction. In 2002, an emergent resection of small intestine and desmoid tumors for intestinal perforation removed only a portion of the diffuse mesenteric disease. This operation was complicated by an enterocutaneous fistula from the jejunum that subsequently healed with conservative care. She had no adjuvant radiation therapy at any point. Estrogen and progesterone receptor expression of the tumors was never assessed.

**Figure 1:**
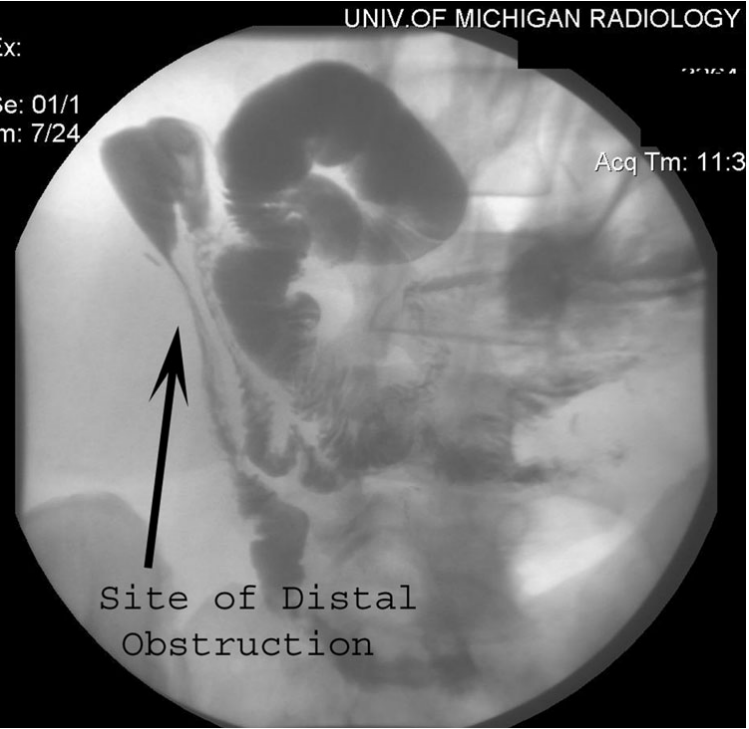
Fistula injection with small bowel follow-through demonstrating distal obstruction of small bowel.

**Figure 2:**
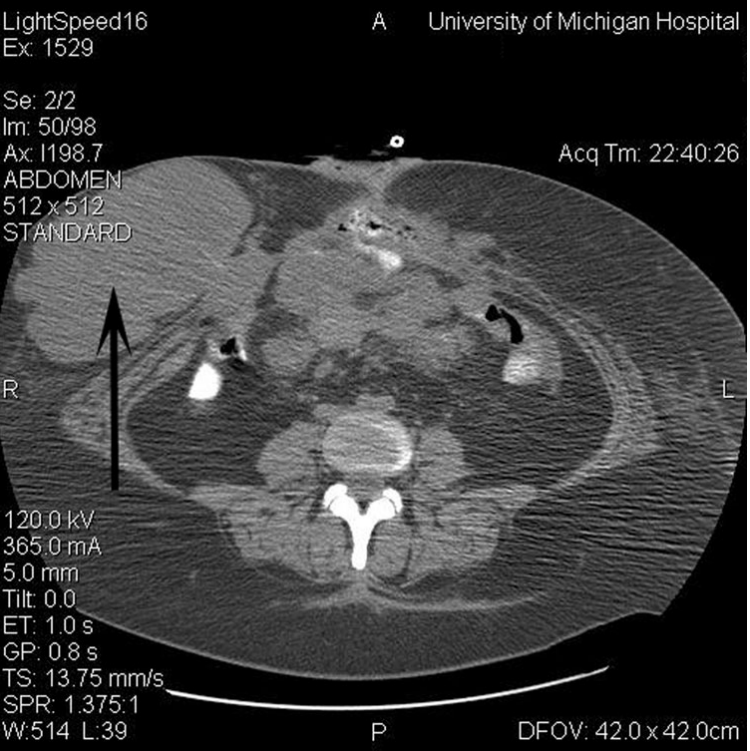
Computed tomogram of the abdomen prior to initiation of cytotoxic chemotherapy demonstrating right-sided abdominal wall soft tissue mass (16 × 9 cm) (arrow), and mesenteric soft tissue mass (7 × 5 cm).

**Figure 3:**
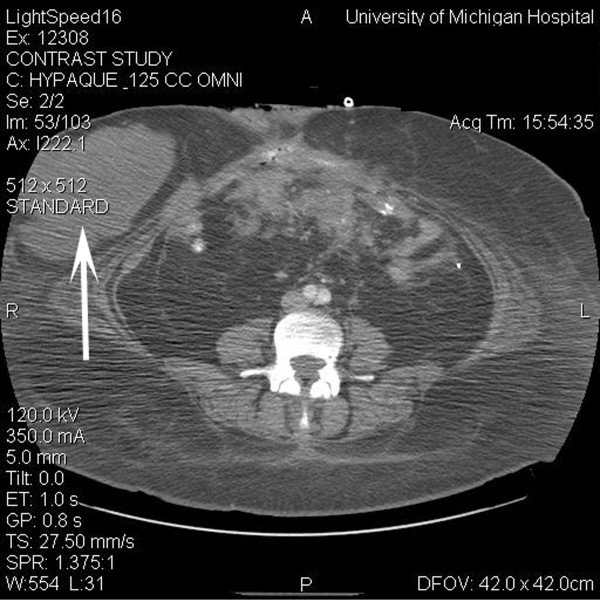
Computed tomogram of the abdomen at the same level as Figure 2 following cytotoxic chemotherapy. The largest abdominal wall lesion decreased to 13 × 7 cm (arrow). Other lesions in the rectus sheath decreased from 6.4 × 3.4 cm and 4.4 × 2.7 cm to 6 × 3 cm and 3.3 × 2.2 cm, respectively. The mesenteric masses decreased markedly.

Despite treatment with anti-inflammatory agents, the patient's abdominal desmoid tumors continued to grow and she was re-admitted 10 months later for recurrence of her enterocutaneous fistula. Radiographic studies showed near-complete small bowel obstruction by desmoid tumor (Figure 1, 2) and complete diversion of the fecal stream through the fistula. She began total parenteral nutrition (TPN). The fistula was not amenable to operative repair without resolution of the distal small bowel obstruction. Options for resolving the distal obstruction included operative resection of the desmoids, a procedure associated with much potential morbidity, or systemic therapy to reduce the size of the desmoids. The patient was offered therapy with anti-estrogen agents or investigational therapy with imatinib. Imatinib was initiated and was ineffective, possibly related to the patient's difficulty with gut absorption. Imatinib was discontinued, and she began a regimen of cytotoxic therapy that is active for sarcoma, including doxorubicin 20 mg/m^2 ^IV on days 1, 2, and 3 and ifosfamide 2 g/m^2 ^daily for 3 days of a 21 day cycle. She received five cycles of this regimen, with a clear radiographic response on CT scan (Figures 2, 3). However, her high-output fistula continued.

**Figure 4:**
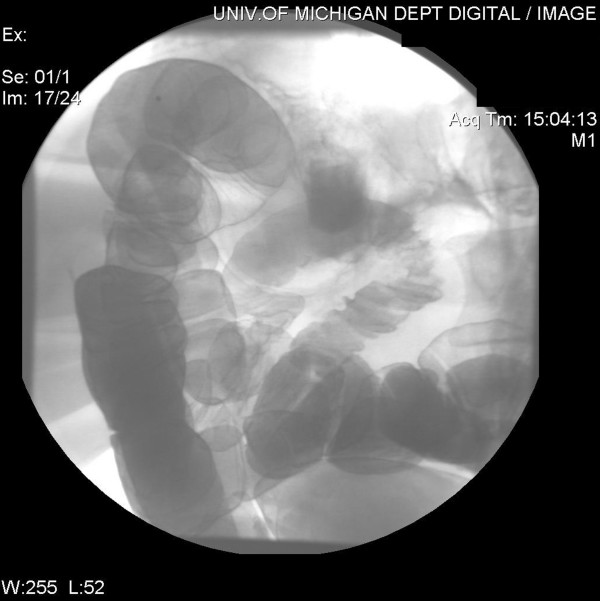
Follow-up barium enema revealing no evidence of distal obstruction and anatomy suitable for segmental intestinal resection of the enterocutaneous fistula. Contrast refluxed from the anus through the previously obstructed area, and out the fistula.

A barium enema (Figure 4) demonstrated resolution of the distal obstruction with reflux of contrast through the fistula. Enterocutaneous fistula resection with primary enteroenterostomy was performed without complication. The patient was discharged on TPN for continued nutritional support, although she was able to tolerate oral feedings with normal bowel function. Her midline surgical wound subsequently healed by secondary intent. With the return of gastrointestinal function, antiestrogen therapy with tamoxifen 20 mg po daily was initiated.

## Discussion

Approximately 10% of patients with FAP develop desmoid tumors as part of their extra-colonic manifestations [1]. Although they are histologically benign and typically slow-growing, desmoid tumors do have the capacity to become locally aggressive. Treatment for desmoid tumor is indicated for symptoms, risk to adjacent structures, or cosmesis. In this case, treatment of desmoid tumor was indicated for small bowel obstruction caused by desmoid tumor growth.

Surgical resection with negative pathologic margins is optimal for treatment of most symptomatic desmoid tumors [2]. Post-operative adjuvant radiation therapy may be useful when macroscopic residual tissue is present, particularly in areas with relatively radiation-tolerant surrounding normal tissues. For patients with large tumors or impediments to complete resection, neoadjuvant management with non-cytotoxic or cytotoxic agents may be of benefit. Intra-abdominal desmoids in particular are often not amenable to margin negative resection, and in some instances cytotoxic chemotherapy has achieved favorable results [3,4] In this case, chemotherapy reduced the size of a tumor causing distal obstruction, allowing bowel resection to resolve an enterocutaneous fistula.

For lesions requiring systemic therapy, hormonal therapy including tamoxifen, toremifine, raloxifene, and progestinal agents have been effective in a subset of patients [5-7]. Non-steroidal anti-inflammatory drugs may be used alone or in combination with tamoxifen [8]. Sulindac is a commonly used NSAID to treat desmoid tumors. Imatinib (Gleevec), a tyrosine kinase inhibitor, has been effective in treatment for desmoid and a phase II multicenter trial investigating the activity of this agent is underway [9,10].

Cytotoxic chemotherapy is indicated when other measures fail. There can be a significant increase in treatment-related morbidity, which may be justified in some clinical settings. Typically, initial treatment is low-dose systemic chemotherapy to limit side-effects. For instance, treatment with methotrexate and vinblastine given every 7–10 days for several months has stabilized advanced and aggressive disease in some patients [11]. However, responses are often slow and generally occur over many months.

More intensive cytotoxic chemotherapy, such as the combination of doxorubicin and dacarbazine, has been effective in patients who have unresectable disease that is refractory to endocrine therapy, steroids and NSAIDs [12,13]. Combination chemotherapy using doxorubicin and ifosfamide produced a relatively rapid radiologic and clinical response in our patient.

Although desmoid tumors have no metastatic potential, they may often be locally aggressive, increasing the morbidity and mortality of patients with Gardner's syndrome. As we gain experience in detecting and treating adenomatous disease early, we can expect the extra-colonic manifestations of Gardner's syndrome to play a greater role in the prognosis of these patients. For desmoid tumors, treatment options extend beyond surgical resection, radiation therapy, and non-cytotoxic systemic therapies and can include cytotoxic chemotherapy to the benefit of our patients.

## Competing interests

The author(s) declare that they have no competing interests.

## Authors' contributions

**GD, LB and RC **treated the patient clinically and designed the study, **PB **and **RC **drafted the manuscript. **PB, RC, LB **and **GD **all contributed to critical revisions and intellectual content. All authors read and approved the final manuscript.
